# Rings in the Extreme: PCNA Interactions and Adaptations in the Archaea

**DOI:** 10.1155/2012/951010

**Published:** 2012-11-14

**Authors:** Jody A. Winter, Karen A. Bunting

**Affiliations:** ^1^Centre for Biomolecular Sciences, University of Nottingham, University Park, Nottingham NG7 2RD, UK; ^2^Centre for Genetics and Genomics, University of Nottingham, Nottingham NG7 2UH, UK

## Abstract

Biochemical and structural analysis of archaeal proteins has enabled us to gain great insight into many eukaryotic processes, simultaneously offering fascinating glimpses into the adaptation and evolution of proteins at the extremes of life. The archaeal PCNAs, central to DNA replication and repair, are no exception. Characterisation of the proteins alone, and in complex with both peptides and protein binding partners, has demonstrated the diversity and subtlety in the regulatory role of these sliding clamps. Equally, studies have provided valuable detailed insight into the adaptation of protein interactions and mechanisms that are necessary for life in extreme environments.

## 1. PCNA Plays a Central Role in the Regulation of DNA Replication and Repair

Sliding clamps, including the proliferating cell nuclear antigen (PCNA), are central to DNA replication processes. These molecules encircle DNA, thereby forming a platform for DNA polymerases to achieve the high processivity required for DNA replication. Additionally, many other molecules involved in DNA processing, such as DNA repair enzymes, DNA modulating enzymes, and other DNA polymerases, bind to PCNA via a conserved motif. PCNA is therefore a crucial factor in determining access to DNA and, hence, is central to regulation, coordinating hand off between enzymes and limiting access of potentially mutagenic translesion polymerases to the replication fork.

## 2. The Structure of PCNA Is Globally Conserved between the Archaea and Eukaryotes

**Figure 1:**
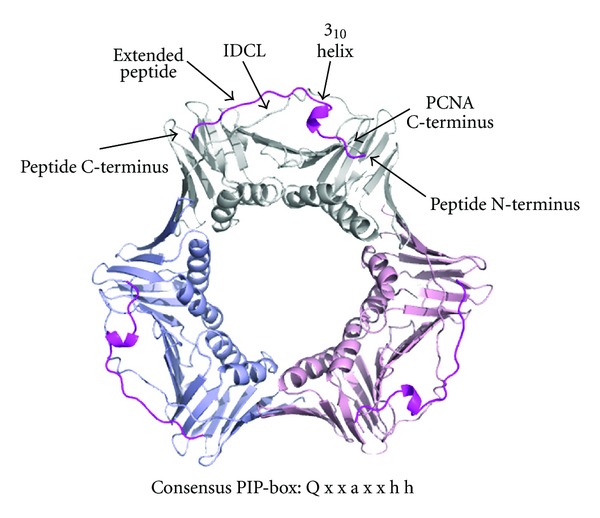
Human PCNA in complex with p21 peptide (1AXC) [2]. PCNA is a trimeric molecule, with each monomer containing a PIP-box binding site and located between the C-terminus of the protein and the interdomain connector loop (IDCL). The human PCNA in complex with p21 shows the classic mode of interaction, with the conserved glutamine of the PIP-box motif interacting both directly and via solvent molecules with the clamp surface. The turn of 3_10_ helix and the immediately C-terminal region insert the hydrophobic section of the motif into the hydrophobic cleft on the PCNA surface. The extended peptide forms an interaction with the IDCL. Each PCNA monomer is coloured separately with the p21 peptide in magenta. Key features are labelled. Also shown is the PIP-box consensus motif, where a indicates a small hydrophobic residue, h is an aromatic residue, and x is any amino acid [1]. Figures, unless otherwise stated, were produced using PyMol [28].

Structural analysis of sliding clamps and their binding partners has proved to be crucial in the elucidation of regulation of both DNA replication and repair processes. PCNA-binding partners generally possess a PCNA-interaction peptide (PIP) motif, usually located at the extreme N- or C-terminus. The PIP-box consensus motif in both eukaryotes and archaea has been defined as Q x x a x x h h, with h representing a small hydrophobic group (Ile/Leu/Met) and a an aromatic hydrophobic amino acid (typically Phe/Trp) [1]. Structural characterisation of human PCNA complexed with a C-terminal peptide from the cell cycle checkpoint protein p21 revealed the role that these conserved residues play in the interactions, which have been subsequently shown to be typical of the bulk of PCNA-PIP-box interactions (Figure 1). The conserved glutamine side chain forms multiple interactions with PCNA surface residues, both direct and water-mediated [2]. A section of 3_10_ helix follows the conserved glutamine, and this section, together with the immediate C-terminal residues, inserts the crucial hydrophobic components of the motif into the pocket on the clamp surface. C-terminal residues lie in an extended conformation, interacting with the interdomain connector loop (IDCL) of PCNA. Residues prior to this glutamine frequently form a section of antiparallel  *β*-sheet with the extreme C-terminus of PCNA.

It is particularly intriguing that this overall mode of interaction is largely conserved in PCNA-interacting proteins. Coupled with the fact that an enormous number of proteins possess PIP-boxes, it is therefore of great interest to determine precisely how hierarchies of interactions occur, allowing temporal and spatial coordination of the multiple binding partners to maintain genome integrity.

The archaea represent a fascinating opportunity to explore these interactions. Not only are PCNAs from the archaea well represented in the Protein Data Bank, but also greater variation is observed in the family than is seen in eukaryotes, as has been noted for other families of DNA-interacting proteins [3]. Comparison of these variations is potentially very insightful in terms of clarifying protein structure-function relationships. The euryarchaeal PCNAs are typical of the family, being homotrimeric in nature as seen in eukaryotes, although examples exist of an euryarchaeon possessing two distinct PCNA rings [4]. In contrast, heterotrimeric PCNAs have been characterised in crenarchaea, with each subunit binding specific partners [5]. Recent work suggests variation in formation of the protein rings that can occur in crenarchaea [6]. This variety is a crucial resource in understanding the mode of action of PCNA and other sliding clamps.

## 3. The Emergence of Modulating Protein-Protein Interfaces

**Figure 2:**
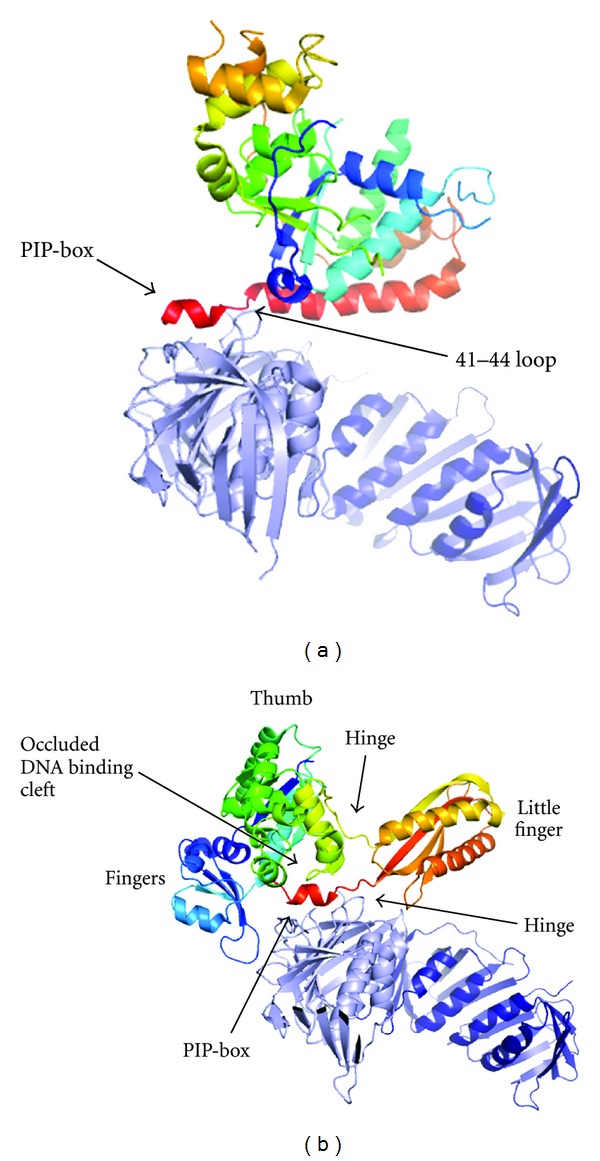
Interactions of the SsoPCNA 1 and 2 heterodimer with (a) Fen1 (2IZO) and (b) Dpo4 (3FDS) [9, 10]. The PCNA subunits are coloured light (PCNA1) and dark (PCNA2) blue. The principal PIP-box interaction is indicated, as are other key interacting domains.

Interest in recent years has focussed beyond the principal PIP-box interaction, on the identification of additional modulating protein-protein interfaces. The existence of such interfaces had long been postulated as a mechanism to permit additional levels of regulation, and the first structural characterisation of a regulatory interface showed the *E. coli* Pol IV translesion polymerase in a “locked out,” inactive orientation on the  *β*-clamp [7]. The complex of human PCNA with Flap endonuclease 1 (Fen1) demonstrated that such interactions existed between that enzyme with PCNA [8], followed in 2006 by the structure of the *Sulfolobus solfataricus* (Sso)PCNA 1 + 2 heterodimer in complex with Fen1 [9]. Beyond the PIP-box interaction, described in greater detail below, the SsoPCNA1 loop 41–44 interacts with the first helical section in Fen1 (Figure 2(a)). Residues Asp-43 and Lys-44 in this exposed loop of PCNA1 form salt bridges with Fen1 residues Lys-17 and Asp-343, thereby contacting both the N-terminus of Fen1 and the PIP-box to maintain the orientation of Fen1 relative to the PCNA ring.

In contrast the structure of the same SsoPCNA 1 + 2 heterodimer in complex with the potentially mutagenic translesion polymerase Dpo4 showed more substantial, presumably regulatory, interaction beyond the PIP-box [10]. Further interactions occur between the finger, thumb, and little finger domains of Dpo4 (Figure 2(b)), as opposed to the single extra interaction seen with Fen1. The conformation of Dpo4 differs from that observed for either the apo-enzyme or the DNA-bound form and is possibly due to the presence of two flexible hinges, either side of the little finger domain. Although the nature of the sliding clamp interactions differ from those seen with the bacterial Pol IV, the resulting PCNA-Dpo4 conformation is similarly not competent for replication, since the binding site is directly involved in interactions with the PCNA surface, effectively blocking productive interaction with DNA.

**Figure 3:**
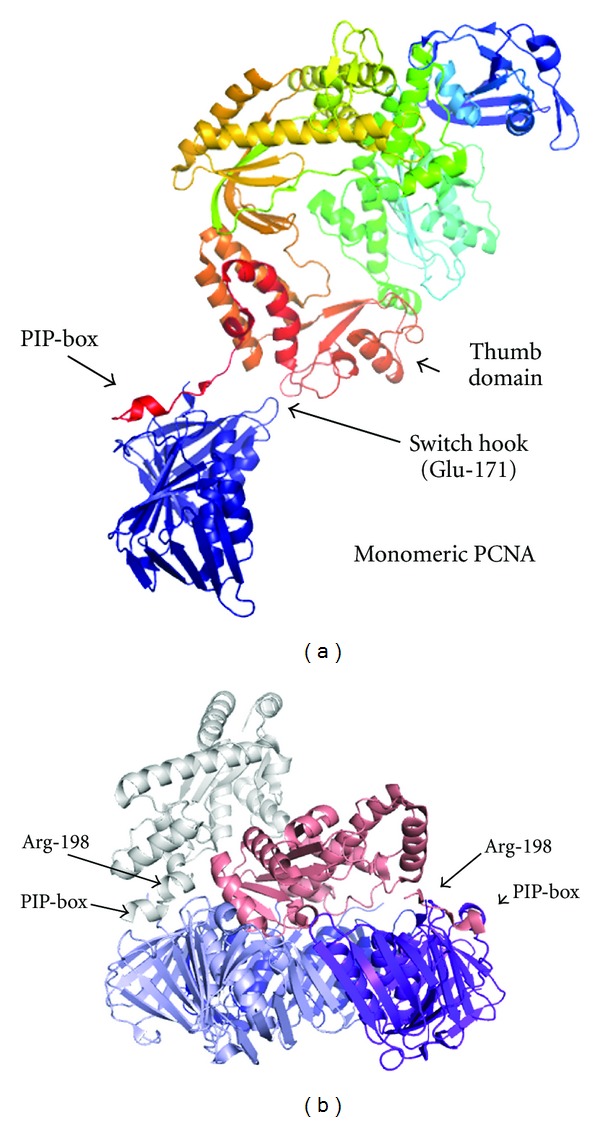
(a) Interaction of PfuPol with a monomeric mutant form of PfuPCNA (3A2F) [29]. The interacting proteins are shown coloured by spectrum from the N-terminus (blue) to the C-terminus (red). (b) Interactions of AfuRNase H with AfuPCNA (3P83). Three protein chains are bound in the structure, for clarity chain D has been removed. The PCNA ring is coloured purple, light, and dark blue. The chain occluding the central pore is coloured in salmon, and one of the two chains oriented away from the pore is coloured grey. The position of the key interacting residue AfuRNase^Arg-198^ is indicated [12].

The structure of the *Pyrococcus furiosus* (Pfu) DNA polymerase B in complex with a monomeric mutant form of the PfuPCNA shows a single additional interaction beyond the PIP-box [11]. A conserved negatively charged residue (Glu-171) on an exposed PfuPCNA loop interacts with Tyr-654 and Arg-706 in the thumb domain of the polymerase (Figure 3(a)). Consequently, the polymerase appears to be in a standby mode between the inactive and active conformations. This “switch-hook” region is therefore postulated to regulate switching between the polymerisation and exonuclease modes of the polymerase, critical in coordinating these linked functions.

The recently solved structure of the *Archaeoglobus fulgidus* (Afu) RNase H complexed with PCNA shows two binding modes in the three bound enzymes, with one molecule occluding the central pore of the sliding clamp and the other two oriented away from the ring [12] (Figure 3(b)). This range of motion is permitted by a hinge region immediately prior to the AfuRNase H PIP-box, containing Arg-198, which appears to play a crucial role in determining the mode of binding. In the extended rotamer, AfuRNase^Arg-198^ interacts with AfuPCNA^Ser-244^, and an additional hydrogen bond is present between AfuRNase^Ser-195^ and AfuPCNA^Arg-241^. In the occluding-mode molecule, the side chain of AfuRNase^Arg-198^ folds back and disrupts this hydrogen bond, allowing a salt bridge to form between AfuPCNA^Arg-241^ and AfuPCNA^Asp-150^. AfuRNase^Arg-198^ is crucial in the functioning of this molecular switch.

## 4. Despite the Degree of Conservation, PIP-Box Binding Shows Hidden Subtleties

Undoubtedly analysis of the PCNA-protein complexes has provided a wealth of information on the role of secondary binding interfaces in regulation. However, comparison of the PIP-box interactions also shows subtle variation that could be critical in the establishment of hierarchies amongst different PCNA-binding partners. Polymerase usage hierarchies have been identified in *E. coli*, and both the  *β*-binding motif and additional modulating protein interfaces have been shown to affect competition between polymerases for clamp binding and access to clamp-associated DNA [13]. 

**Figure 4:**
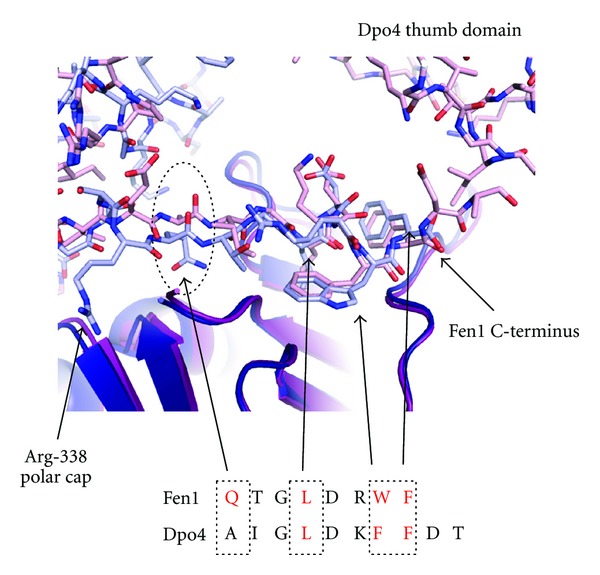
Detailed comparison of the PIP-box interactions of SsoFen1 and Dpo4 with PCNA (see also Figures 2(a) and 2(b)). Fen1 and PCNA1 are shown in light and dark blue, respectively. Dpo4 and its respective PCNA1 are shown in pink and magenta. The position of the conserved glutamine residue is highlighted and the key residues in the interaction motif are indicated in red (2IZO and 3FDS, [9, 10]). Residues in the top right hand corner are part of the Dpo4 thumb domain and are proximal to the extreme C-terminus of the protein.

Comparing the PIP-box-mediated interactions of Fen1 and Dpo4 with SsoPCNA shows that the Sso Fen1 PIP-box lacks interaction with the IDCL, since the terminal residue of the motif represents the extreme C-terminus of the protein (Figure 4). In contrast, the two additional residues at the C-terminus of Dpo4 extend towards the thumb domain of the enzyme, although no interaction is observed with the IDCL. The region prior to the conserved glutamine in Fen1 forms a  *β*-zipper of antiparallel  *β*-sheet with the C-terminus of PCNA. This is topped by a polar cap of Arg-338 contacting the clamp surface, compensating for the loss of the IDCL interaction.

Dpo4 lacks the conserved glutamine of the PIP-box motif present in Fen1, although the peptide backbone conformation is very similar in this region. It has been previously noted in *E. coli* and humans that translesion polymerases tend to show greater deviation from the canonical binding motifs, with higher levels of conservation presumably reflecting a requirement for tighter binding in replicative polymerases [7, 14].

**Figure 5:**
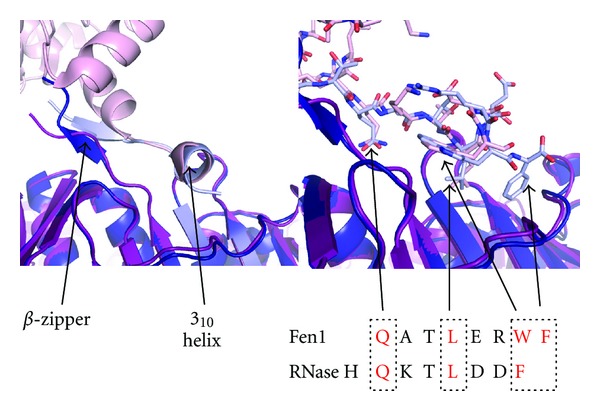
Detailed comparison of the PIP-box interactions of AfuPCNA with a Fen1 peptide (1RXZ) and RNase H (3P83) [12, 15]. The left hand panel depicts the binding partners in cartoon representation and the right hand panel in stick form. The Fen1 complex is shown in light/dark blue and the RNase H complex in pink/purple. Key features and conserved residues in the PIP-box are indicated.

Comparison of these two AfuPCNA complexes also reveals variation in the region preceding the PIP-box. The AfuFen1 PIP-box peptide complexed with PCNA possesses a  *β*-zipper region, linking the PIP-box with the adjacent DNA-binding region of Fen1 (Figure 5). A conserved proline residue (Pro-240) serves to direct this zipper region outwards. This alters the position of the PCNA C-terminus and results in enhanced binding of Fen1 to DNA, permitting communication between the PIP-box and DNA-binding regions [15].

The regions immediately prior to the PIP-box (the  *β*-zipper in the case of Fen1 and Arg-198 for RNase H) appear to compensate for the lack of IDCL interactions. The interactions maintained by AfuRNase H Arg-198 are likely to be more crucial given the lack of the second aromatic residue in the RNaseH PIP-box.

## 5. Adaptation under Extreme Conditions

Whilst archaeal PCNAs have proven highly informative in the characterisation of binding and regulatory modes that can be extrapolated to eukaryotic systems, the structures have also highlighted intriguing complexity in terms of adaptation to the often extreme environments inhabited by archaea. These environmental conditions pose particular challenges to proteins which must function under, for example, extremely high temperature or salt conditions. Typically proteins display overall conservation of their architecture with their mesophilic counterparts, with changes to compensate for their lifestyle, described in more detail below.

The PfuPCNA structure, the first archaeal PCNA solved, is a classic example of protein adaptation to high temperatures [16]. Comparison with the yeast and human structures showed the hyperthermophilic PCNA to have a reduction in the proportion of polar uncharged residues and an increase in charged residues, consistent with the trend in thermophiles for higher numbers of ion pairs. These adaptations are thought to contribute to protein stability [17], and PfuPCNA is no exception, with 10 of the 27 ion pairs present in the crystal structure being intermolecular in nature, increasing the stability of the trimer. Differences in loop length mean that PfuPCNA has fewer hydrogen bonding interactions across the monomer-monomer interface compared with mesophilic PCNAs, with this presumably compensated for by the increase in ionic interactions. Intriguingly, binding to the PIP-box peptide from the Replication Factor C, large subunit appears to stabilise the PfuPCNA trimer via a domain shift in the C-terminal domain, resulting in increased numbers of hydrogen bonds at the monomer-monomer interface and a rearrangement in the ion pairs [18].

**Figure 6:**
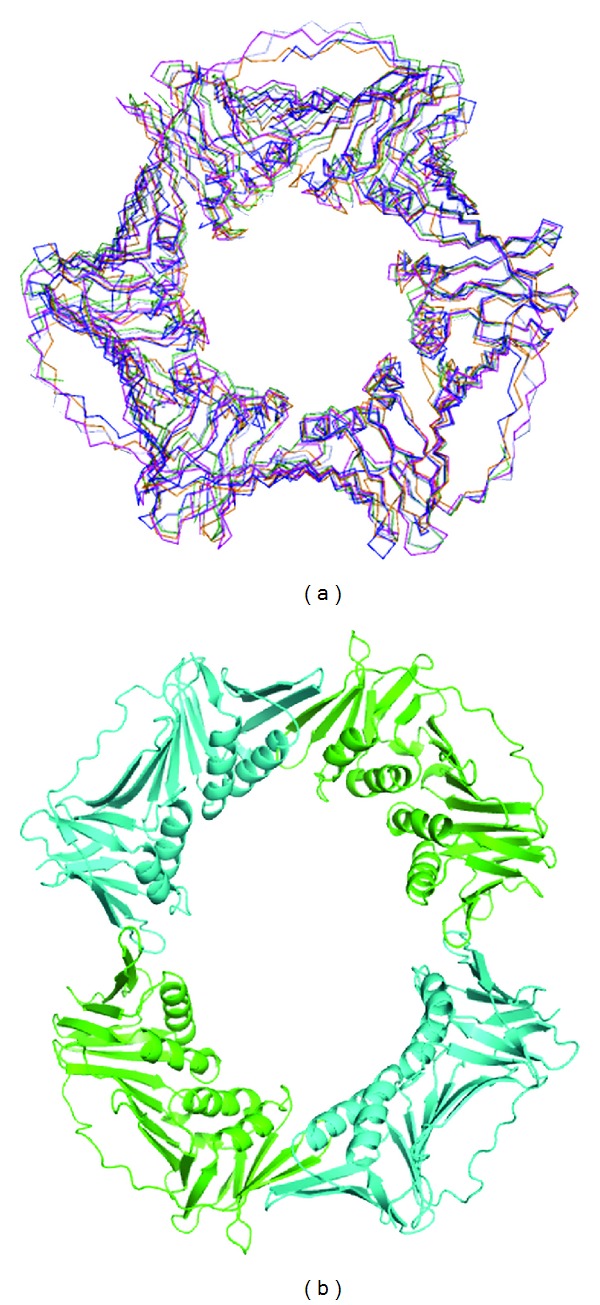
Global architecture of the archaeal PCNAs. (a) Superposition of the characterised homotrimeric PCNAs showing the high conservation of secondary and tertiary structure. HvoPCNA (blue-3IFV); PfuPCNA (green-1GE8); Tko PCNAs (light blue/magenta—3LX1/3LX2); AfuPCNA (orange-1RWZ). (b) The heterotetrameric structure of *Sulfolobus tokadii* PCNA 2 and 3 (3AIZ) [6].

Subsequent structures of homotrimeric archaeal PCNAs show that they retain the same overall architecture and disposition of secondary structural elements (Figure 6(a)). However, the situation is more complicated in the crenarchaea, a recent study of the heterotrimeric *Sulfolobus tokodaii* PCNA shows that the PCNA 2 and 3 subunits can form a heterotetramer both within the crystal and in solution (Figure 6(b)). The authors postulated that the flexibility of ring formation in the heterotetrameric PCNA would permit this form to function as a Holliday junction clamp [6]. Variation in subunit composition of heterotrimeric PCNAs has been previously reported [19]. Such flexibility may prove advantageous in permitting multiple alternative forms of PCNA, each optimal for a particular cellular function.

Superposition of the monomer-monomer interfaces shows some variation in loop regions adjacent to the intermolecular  *β*-sheet. The *Haloferax volcanii* (Hvo) PCNA and AfuPCNAs have truncated interfaces compared with PfuPCNA [20]. In contrast, *Thermococcus kodakarensis* (Tko) unusually has two PCNA-encoding genes, and the structures of the two gene products have recently been solved [4]. Each gene product forms a separate trimer. The principal difference between the two proteins lies in their subunit interfaces, affecting the resulting stability of the rings. These variations in stability presumably have a biological function, with one of the rings postulated to have been acquired through lateral gene transfer; both were demonstrated to be capable of stimulating polymerase activity. Archaeal PCNAs are thought to be capable of self-loading onto DNA, unlike their eukaryotic counterparts and self-loading, followed by the stabilising effects of PIP-box binding observed in PfuPCNA may prove advantageous to archaea, where extreme conditions can result in a higher rate of DNA damage and a requirement for enhanced efficiency of DNA repair processes [18, 21].

**Figure 7:**
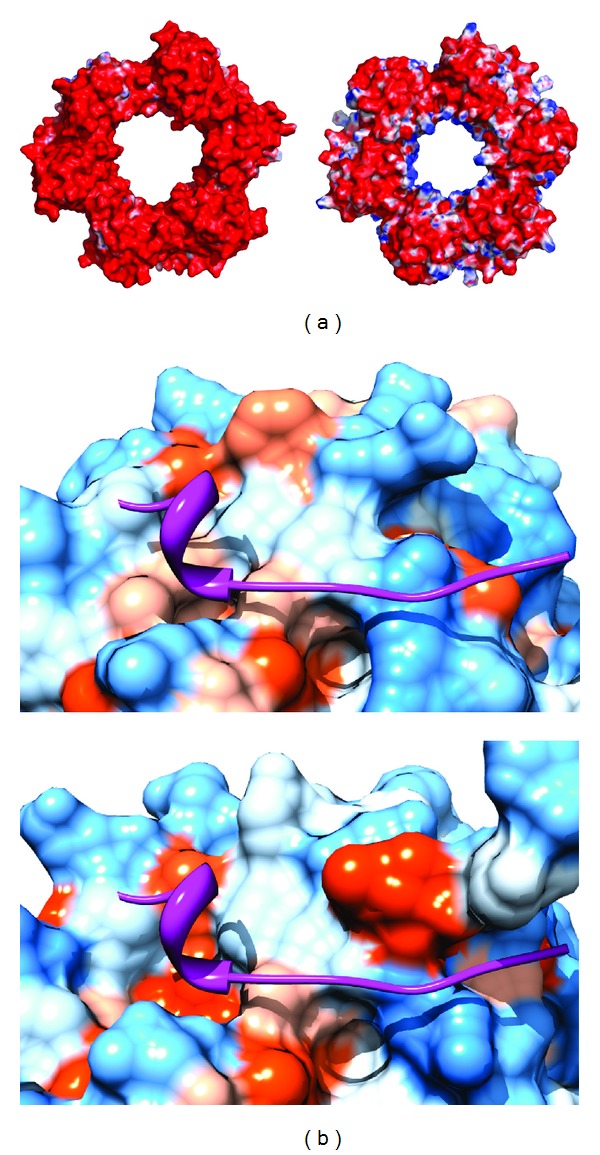
Adaptation to the high salt environment of HvoPCNA [20]. (a) Electrostatic surface representation of HvoPCNA (left—3IFV) and the more typical AfuPCNA (right—1RWZ), with the surface coloured from  −10*k*_*B*_ *T*/*e*  (red) to  +10*k*_*B*_ *T*/*e*  (blue), showing loss of the electropositive charge from the central pore of PCNA are calculated using APBS [30]. (b) The HvoPCNA binding pocket (top) shows a reduction in hydrophobic character and in depth of the binding pocket compared to AfuPCNA (bottom). The surfaces are coloured according to the Kyte-Doolittle scale with blue indicating the most hydrophilic residues through to orange for the most hydrophobic residues. The AfuFen1 peptide of 1RXZ is shown in magenta in both panels for comparison. Produced using Chimera [31].

The majority of archaeal PCNA structures are from thermophilic sources. The HvoPCNA, from a halophile, shows unique adaptation to high salt concentration, with intracellular salt levels in *H. volcanii *approaching saturation [20]. Although the global architecture of HvoPCNA is very typical, the surface charge characteristics are quite distinct compared with other archaeal and eukaryotic PCNAs (Figures 6(a) and 7(a)). Every other sliding clamp has acidic surface characteristics, aside from the electropositive pore. Consistent with the typical reduction in halophiles of lysine residues and an increase in negatively charged residues, HvoPCNA has almost entirely lost this electropositive pore. Instead it appears to harness cations to reduce repulsion effects between its own negative surface and the negatively charged phosphate backbone of the DNA it encircles [20].

Variation is also seen in the HvoPCNA PIP-box binding pocket, which is considerably more shallow than typical, although modelling suggested the general mode of binding is conserved (Figure 7(b)). This reduction in hydrophobic character both in the PIP-box motifs of HvoPCNA binding partners (e.g., the second phenylalanine is substituted for a less bulky hydrophobic residue in the Hvo polymerase PIP-box motifs), and on the surface of PCNA, is consistent with an exaggeration of hydrophobic effects in high salt conditions [20].

**Figure 8:**
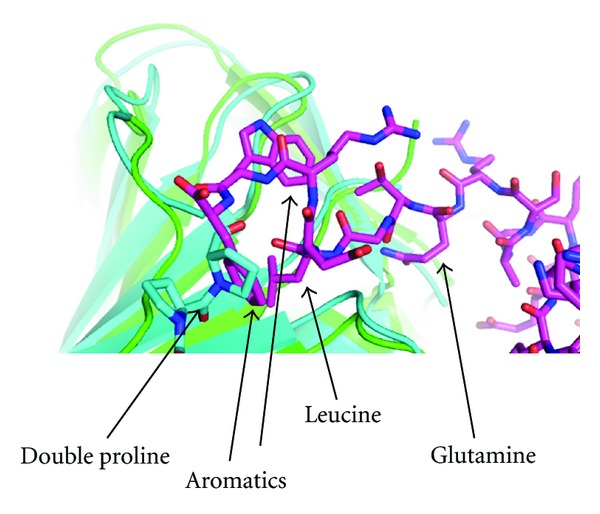
Comparison of the binding pockets of SsoPCNA 1 and 2. The PCNA2 subunit (blue) is superimposed on the PCNA1 subunit of 2IZO (green) to demonstrate that the typical binding motif of Fen1 (magenta, stick representation) cannot be accommodated within the binding pocket of PCNA2 due to steric hindrance [9]. The double proline motif of PCNA2 responsible for the blocking shift in the IDCL is indicated.

The heterotrimeric PCNAs of the crenarchaea represent a unique opportunity to study adaptation of the binding pockets, since binding partners interact specifically with one of the three alternative PCNA molecules, as demonstrated in SsoPCNA [5]. The structure of the SsoPCNA 1 and 2 heterodimer and Fen1 clearly demonstrated the mechanism by which binding of the Fen1 motif is precluded in PCNA2 (Figure 8) [9]. The Fen1 PIP-box is a good fit with the consensus sequence. A shift in the IDCL position initiated by a double proline motif in PCNA2 restricts the binding pocket such that the Fen1 terminal phenylalanine is sterically precluded from binding. The authors concluded that PCNA could accommodate a motif more reminiscent of that seen in *E. coli*, with a consensus of QL(S/D)LF [9, 22], since the PolB1 polymerase, known to bind PCNA2, has a PIP-box motif of QLTLF. Additionally Glu-156 in the PCNA1 polar cap is replaced by valine in PCNA2, which again does not favour Fen1-PCNA2 binding. These rather subtle differences impose a spatial interaction hierarchy not seen in homotrimeric PCNAs. *Sulfolobus* species experience an increased rate of DNA damage due to their extreme lifestyle [23], and increased organisation of DNA-processing enzymes around the PCNA ring could represent an evolutionary advantage for organisms requiring more efficient DNA repair mechanisms due to higher levels of DNA damage.

## 6. Evolution

The organisation of the archaeal PCNAs is highly intriguing, given that it is, in many cases, considerably more complex than in eukaryotes [24]. Detailed phylogenetic analysis suggests that the evolutionary dynamic between archaeal and eukaryotic proteins is very different, despite the high degree of structural conservation [25]. Whilst in eukaryotes the PCNAs appear to have evolved from a common ancestor, this was found to vary between taxons within the archaea. The situation is particularly complex in the crenarchaea, and this analysis showed deep evolutionary branching between the subunits.

What benefit does this complex organisation of alternative heterotrimeric PCNAs offer to the crenarchaea? The strong degree of structural conservation and essential role of PCNA in cellular metabolism is testament to the high level of selective pressure on the functional form. The specific nature of partner binding observed in SsoPCNA expands the options for spatial regulation in the sequential events of DNA replication and repair processes, beyond what is possible in homotrimeric PCNAs. The well-studied example of Fen1, Pol, and ligase known to bind PCNA 1, 2, and 3, respectively, imposes chirality in the sequential actions of these three enzymes in Okazaki fragment maturation [9]. Potentially this results in a loss of flexibility over homotrimeric PCNAs, which must impose temporal and spatial regulation in an alternative mode, perhaps by greater emphasis on secondary modulating interactions. It has been noted that organisms such as *S. solfataricus* are likely to be under high selective pressure to have optimised DNA repair processes [24].

## 7. Conclusions

Many of the archaea thrive in and/or require extreme conditions for survival, and this undoubtedly is a key contributor to the high level of variation seen in PCNA subunit structure and multimeric composition. In line with the observation that trends of adaptation exist rather than strict rules, the known structures vary in modes of adaptation. As discussed, the hyperthermophile *S. solfataricus* has adopted a heterotrimeric approach to PCNA adaptation. In contrast the thermophilic lifestyle of *P. furiosus* has resulted in adaptation of the subunit interface and an increase in ion pairs. Whilst the optimal temperature for growth of *A. fulgidus* is somewhat lower, it still grows between 60 and 95°C, yet its PCNA subunit interface is more similar to that of HvoPCNA, which grows between 30 and 50°C [26, 27]. That protein in turn shows unique adaption in its surface charge characteristics and ion binding to compensate for the organism's hypersaline lifestyle. The two separate PCNA trimers of *T. kodokarensis* presumably both have essential functions in the cell, with one likely acquired by lateral gene transfer and possibly viral in origin [4]. The heterotetrameric structure of *S. tokodaii* hints at a further level of flexibility in organisation and will doubtless prompt further discoveries [6].

The overall mode of PIP-box interaction is conserved across the characterised archaeal interactions, with the greatest deviation, perhaps predictably, occurring in the heterotrimeric PCNAs. Since the motifs generally reside at the extreme C-terminus of the binding partners, little interaction is seen between the peptides and the PCNA IDCL, as observed in some eukaryotic complexes. Variation is greatest in the region immediately prior to the PIP-box, reflecting the varied function of the binding partners. As might be expected, the widest variation is seen at points of additional contact beyond the PIP-boxes, and these interactions are likely to play crucial roles in defining the spatial and temporal handoff between binding partners, ensuring the maintenance of genome stability.

These comparisons furnish us with a fascinating insight into how far proteins can be pushed whilst functioning in the cell in a role conserved from phage through to humans. Equally, they demonstrate how profound a difference a single amino acid substitution can make. Understanding these adaptations can only enhance understanding of fundamental biology as well as providing an essential platform for structure solution of complexes that give real insight into the regulation of key processes in eukaryotes that are essential for genomic stability.
